# Human Gut Microbiota Plasticity throughout the Life Course

**DOI:** 10.3390/ijerph20021463

**Published:** 2023-01-13

**Authors:** Kerstin Thriene, Karin B. Michels

**Affiliations:** 1 Institute for Prevention and Cancer Epidemiology, Faculty of Medicine and Medical Center, University of Freiburg, 79110 Freiburg, Germany; 2 Department of Epidemiology, Fielding School of Public Health, University of California, Los Angeles, CA 90095, USA

**Keywords:** human gut microbiota, plasticity, exposome, window of opportunity

## Abstract

The role of the gut microbiota in human health and disease has garnered heightened attention over the past decade. A thorough understanding of microbial variation over the life course and possible ways to influence and optimize the microbial pattern is essential to capitalize on the microbiota’s potential to influence human health. Here, we review our current understanding of the concept of plasticity of the human gut microbiota throughout the life course. Characterization of the plasticity of the microbiota has emerged through recent research and suggests that the plasticity in the microbiota signature is largest at birth when the microbial colonization of the gut is initiated and mode of birth imprints its mark, then decreases postnatally continuously and becomes less malleable and largely stabilized with advancing age. This continuing loss of plasticity has important implication for the impact of the exposome on the microbiota and health throughout the life course and the identification of susceptible ‘windows of opportunity’ and methods for interventions.

## 1. Introduction

### 1.1. Human Gut Microbiota and Host Interactions

Increasing research reports a great complexity of the human gut microbiota containing a vast number of microorganisms including bacteria, archaea, protists, fungi, and viruses. While the focus remains on studying the bacterial component of the gut microbiota, an increasing number of studies target the other microorganisms: viruses that infect bacteria, bacteriophages, have received more attention in recent years, as they can not only be used in phage therapy to fight pathogenic bacteria, but studies also indicate that they can contribute to disease progression as a permanent component of the human gut microbiota [1]. Fungi, which have garnered heightened attention as well, were shown to be part of the human gut microbiota and may affect the immunological responses of the host [2]. While the microbiota is defined as a community of microorganisms living in a specific environment, such as the human gastrointestinal tract, the microbiome describes the whole entity of all the genomic elements of the microbiota and can also be described as the metagenome of the microbiota [3]. Commensal bacteria of the human gut microbiota are crucial to maintaining host homeostasis by protecting against pathogens, training the immune system, and assisting with nutrient uptake and processing of dietary compounds such as vitamins while the human host serves as a habitat for its microbiota providing nourishment through ingested food [3,4,5,6]. This strongly interwoven alliance between human host and its microbiota is described as a mutualistic relation and some even consider the microbiota a human organ [7,8,9].

The microbiota is malleable by environmental exposures in particular diet, other lifestyle factors, and use of drugs such as antibiotics summarized as the ‘exposome’ [10,11,12,13,14]. Nutritional compounds that cannot be metabolized by the human digestive enzymes in the small intestine but can serve as substrates for bacteria in the colon are referred to as prebiotics. Prebiotics can be dietary fibers that are a source of complex polysaccharides whose glycosidic linkages can be cleaved by specialized bacterial enzymes in the colon leading to monosaccharides that then can be fermented into short-chain fatty acids (SCFAs) [15]. SCFAs such as acetate, propionate or butyrate can be absorbed by the epithelial cells in the colon. Those enterocytes metabolize most of the butyrate directly. Large amounts of propionate are metabolized in the liver while the majority of the acetate is transported to the periphery via the blood stream [16]. Latest research suggests that SCFA uptake results in a wide range of health benefits for the human host including stabilization of glucose homeostasis and blood lipid profiles as well as reduced body weight and lower colon cancer risk [17]. SCFAs, especially butyrate and propionate, also influence host immunity [18,19] via inhibition of histone deacetylases (HDAC) [20,21]. This promotes hyperacetylation of histones in T-cells leading to their differentiation into effector (such as Th1 or Th17 cells) and regulatory T cells (such as interleukin-10 cells) [22], indicating that microbiota in the human gut can affect the hosts’ immune homeostasis by acting as an epigenetic regulator [21].

An increase in inflammatory markers is observed with advancing age. The slow deterioration of the immune system with aging, also called ‘immunosenescence’, causes rise of morbidity and mortality in older individuals due to an increasingly inefficient adaptive immune response to a variety of infections. This chronic, low-grade, systemic inflammation occurring with advancing age is also referred to as ‘inflammaging’ [23,24]. Thymic involution, starting from adolescence and lasting to age 40 to 50 years, leads to less naïve T-cells and more effector and regulatory and memory T-cells, and an increased secretion of pro-inflammatory cytokines such as interleukin 6 (IL-6), tumor necrosis factor alpha (TNF-α), and C-reactive protein (CRP) leading to an elevated inflammation level and age related chronic health conditions [23,25,26]. Studies of germ-free mice have revealed that immune cell development was induced after exposure to certain bacteria derived from the gut microbiota [27,28]. This complex host–microbiota interaction supports the idea the human gut microbiota might have crucial influence on host immune homeostasis by induction and training of the host immune system, and is therefore crucial in maintaining host health [29].

### 1.2. Determinants of the Microbial Signature

In order to define a ‘normal’ or ‘healthy’ gut microbiota, several large-scale studies applying next generation sequencing methods were conducted to gain more insights into the complex mechanisms of the human gut microbiota, helping to define a ‘core microbiota’ [4,30,31,32,33]. Still, a definition of a concrete dataset displaying these traits is challenging, as there are vast interindividual differences between the microbiota of apparently healthy people. Numerous factors, such as genetics, environment, and host habits, such as personal dietary habits and physical activity levels, influence microbial composition to widely varying degrees and have a greater or lesser impact on health outcomes [34,35].

Host genetics influence to some extent gut microbial composition due to mutations in various genes [36,37]. People carrying mutations in the nucleotide-binding oligomerization domain-containing protein 2 (NOD2) gene display an increased abundance of *Enterobacteriaceae* [38]. To investigate associations between host genetics and gut microbiota on a broader basis, Rothschild et al. analyzed data from the Twins UK cohort including more than 14,000 twins to date and concluded that the influence of the environment strongly exceeds that of host genetics in the development of the human gut microbiota. They observed an average heritability of gut microbiota taxa of less than 2%, while over 20% of the interindividual differences of the microbiota were related to diet, drugs and anthropometric measurements [39].

Individual habits, such as dietary patterns have a major impact on the microbial composition by inducing major compositional shifts, as the timing of food intake, for example, can be influenced by circadian rhythm and seasonal variations. Moreover, shifting from a low-fat, high-fiber diet to a high-fat, high-protein, low-fiber diet leads to decreased species diversity in individual hosts (decrease in α-diversity) but also increases differences in microbial patterns between different individuals (increase in β-diversity) [40]. At the same time, certain body sites harbor different and unique microbial assemblages that must be taken into account during analysis [41]. Additionally, the composition of the human gut microbiota is influenced by the amount of physical exercise depending on the type of exercise as well as the intensity of the physical activity [42,43].

### 1.3. Adaptation of the Human Gut Microbiota to Environmental Factors through Varying Plasticity

The close interaction between the microbiota and the human host that has developed over the course of human evolution exhibits a sensitive homeostasis, and its disruption by intrinsic or environmental influences, termed dysbiosis, can lead to severe disorders in the host [44]. Dysbiosis of the gut microbiota is associated with a broad range of symptoms including the development of cardiovascular disease [45], Parkinson’s disease [46], Alzheimer’s diseases [47], asthma and allergy [48,49], low-level inflammation such as obesity and type 2 diabetes [50,51,52,53], but also with chronic inflammatory diseases of the gastrointestinal tract such as inflammatory bowel diseases (IBD) [54,55,56,57]. In general, multimorbidity with advancing age is associated with an imbalance of the microbiota [43]. Studies in individuals reaching exceptional old ages in good overall health suggest that maintaining a beneficial microbial composition throughout the life course improves survival opportunities for the human host [58,59].

In a rapidly changing environment, a rather rigid composition of the gut microbiota could also be of great disadvantage. Adaptation to highly varying external stimuli is of vital importance for the human host. It ensures protection from environmental insults and an optimized utilization of nutritional components from available food sources to create optimal conditions for host and microbiota. The human host, living in a mutualistic interaction with its individualized microbial community, is able to adapt to a constant changing exposome in a more flexible manner, assisted by a vast number of different bacteria in the gut microbiota that provide greater genetic richness and increased plasticity compared to the more limited human gene set alone [19,60].

Plasticity in a developmental aspect describes a process that shapes a life trait through environmental exposures [61] The term ‘developmental plasticity’ is also used specifically to refer to changes in response to external stimuli, with most of these connections being established from birth through early childhood [62]. The plasticity of the microbiota seems to display a similar pattern, allowing for an appropriate response up to a certain threshold of exposure variation; if this threshold is exceeded, disease can result. Throughout the life course, the plasticity of the microbiota rapidly declines and provides diminishing shielding against environmental insults. Plasticity of the microbiota occurs on different time scales, ranging from daily oscillations of the microbial composition due to wake/sleep cycles and feeding/fasting periods [63,64,65] to adaptations to environmental effects throughout life. The following section describes the different life stages, starting from the fetus in the womb to advanced age, highlighting the plasticity of the gut microbiota at each stage.

## 2. Plasticity of the Human Gut Microbiota throughout the Life Course

### 2.1. Prenatal Period

Throughout the intrauterine life of the fetus, there is a constant exchange between maternal and fetal factors, mainly through the placenta and amniotic fluid. The debate about whether a transfer of maternal bacteria to the fetus occurs or whether the womb is sterile has been ongoing for about a decade. A ‘sterile womb’ paradigm has been initially proposed suggesting initial colonization may start during/right after birth, depending on the mode of delivery [66]. Microbes from the mother are acquired (vertically) and from the environment/community (horizontally) [67].

However, more recently, this paradigm has been challenged by the ‘in utero colonization’ hypothesis, which suggests that the womb harbors its own microbiota and the colonization of the gut begins in utero when several studies detected bacterial, viral or fungal DNA in the womb [67,68,69,70,71,72,73]. In contrast, most recent studies were not able to detect an existing microbiota in the womb and suggest detection of DNA from microorganisms in meconium and amniotic fluid in previous studies occurred due to contamination during the sample collection process or false-positive results in the data analysis pipeline [74,75,76,77]. In a recent study that included two cohorts with a total of over 500 women comparing complicated and uncomplicated pregnancies, de Goffau et al. demonstrated that the human placenta does not harbor microbiota [78]. Nevertheless, they detected DNA of *Streptococcus agalactiae*, which is a main cause of neonatal sepsis, in approximately 5% of samples concluding a minor chance of perinatally bacterial infection in an otherwise overall sterile womb.

In several recent animal studies, no consistent evidence of in utero colonization was detected in placental tissues of rhesus macaques [79], in the placental and fetal tissues of mice [80], or in liver, spleen, or brain cortex from fetal sheep [81]. However, in a recent study, Bi et al. applied a multi-omics approach to analyze cecal content of lambs born through aseptic caesarean section and indicate an in utero microbial colonization of the prenatal fetal gut. They describe a low abundant prenatal fetal gut microbiota with low diversity but presumably metabolically active microbiota, as metabolome analysis revealed a presence of several microbial metabolites including SCFAs [82].

Currently, it is not conclusively clarified whether healthy human wombs are colonized by microorganisms and whether the formation of a human gut microbiota is initiated in utero. Analyses of an in utero microbiota remain challenging due to presumably low abundance of microbes and the analytical limitations of 16SrRNA sequencing. Hence, more studies are needed including metagenomics, transcriptomics, proteomics, and metabolomics analyses, but also including additional methods such as culturing, histology, or fluorescence in situ hybridization (FISH).

### 2.2. Perinatal Period

A window of highest susceptibility of the microbiota to be imprinted by the environment is at the time of delivery of the fetus. A large number of studies have addressed the microbial pattern that is established depending on the mode of delivery and differs substantially between vaginal delivery or caesarian section [75,83,84]. After vaginal delivery, mainly maternal vaginal microbes colonize the infant’s gut, leading to an enrichment of *Bifidobacterium* spp. and a reduction in *Enterococcus* and *Klebsiella* spp. compared to delivery by caesarian section, in which the infant’s gut is mainly colonized by skin microbes [85]. Caesarean section delivery affects the transfer of microbiota components from the mother to the newborn during a critical window for neonatal immune system priming [86,87]. The difference in microbial composition induced by mode of birth is maintained throughout the neonatal period (up to 28 days after birth) lasting into infancy (up to one to two years after birth) [88]. How long this state is maintained remains unclear. Some studies suggest differences have largely faded by one year of age [89]. Since a prospective cohort study of women participating in the Nurses’ Health Study II reported that cesarean delivery may lead to an increased risk of obesity and type 2 diabetes later in life, it would be of great interest to investigate whether the gut microbiota might act as a mediator [90]. A recent study using a mouse model suggests that Western diet and exercise in early life (from weaning to 6 weeks of age, reaching sexual maturity) have effects on the microbiota that persist into adult life [91].

Gestational age also affects the intestinal microbiota of the fetus. Prematurely born babies have fewer microorganisms in the gut, lower levels of *Bifidobacterium* spp., and higher abundance of pathogens, as well as a diminished barrier function in the colon, leading to increased permeability and thus systemic inflammation [92]. Other important factors affecting the microbiota during the perinatal phase include exposure to antibiotics, either through maternal ingestion or direct exposure of the newborn to antibiotics. This delays the colonization of *Bifidobacterium* spp. and also prolongs recolonization the longer the antibiotic treatment lasts [92]. Zou et al. suggest that intrapartum antibiotic prophylaxis increases dysbiosis of maternal and neonatal microbiota, resulting in lower levels of *Lactobacillus* spp. [93]. Early infections may also impact the newly establishing microbiota, as the immune system of the premature infants is not yet mature and the barrier function of the gut epithelium in the gastrointestinal tract is still evolving. This can lead to systemic inflammation or sepsis, as bacteria from the gut can enter the bloodstream [94,95].

Maternal diet during gestation as well as birth weight of the fetus determine whether and to what extent an individual is susceptible to a variety of diseases such as cardiovascular disease and hypertension later in life. Barker described this concept as the fetal origins of adult disease, which is also known as perinatal programming or ‘Developmental Origins of Health and Disease’ (DOHaD) [96,97,98,99]. Epidemiologic associations between deficient fetal and infant growth and the development of type 2 diabetes and metabolic syndrome later in life have been termed ‘the thrifty phenotype hypothesis’, according to which poor nutrition in early life results in irreversible changes in glucose–insulin metabolism [100]. Because a nongenetic association between low birth weight and type 2 diabetes has been observed [101], this suggests the involvement of epigenetic mechanisms as well as an important role of the gut microbiota [102].

**Figure 1:**
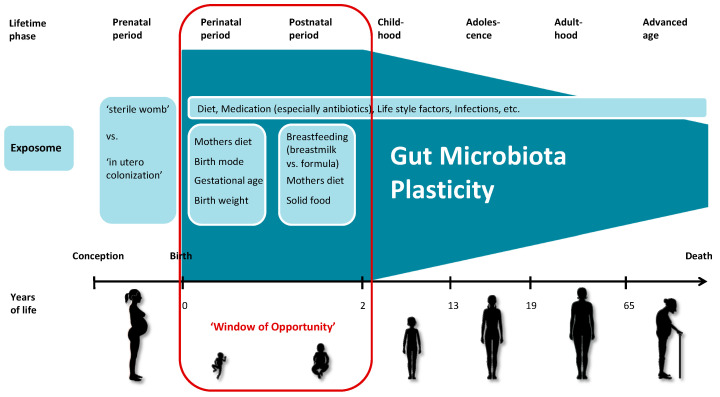
Simplified schema of the plasticity of the human gut microbiota throughout the life course. The microbiota is permanently imprinted by the exposome and subsequently functions as marker for the exposome. The exposome has the greatest potential to determine the microbial composition during the first two to three years of life, which are referred to as the ‘window of opportunity’ in this model. Throughout the life course, the microbiota becomes more stable; it largely returns to its previous state after transient environmental influences. The increase in stability leads to a decrease in the plasticity of the human gut microbiota. (The figure was produced using Servier Medical Art.)

Analyzing data from the Baby Biome Study, a large-scale UK birth cohort study and biobank, Shao et al. describe that the gut microbiota of newborns exhibits strong interindividual variability. When comparing multiple samples from each individual, they also found considerable variation in the composition of the gut microbiota (intraindividual variability), especially during the neonatal period, suggesting that the gut microbiota is highly individualized and very dynamic in the first weeks of life [88]. During this time, the exposome has a major and lasting impact on the developing microbiota, as the mode of delivery, use of antibiotics and geographic location strongly influence microbial composition [103]. In order to adapt quickly and successfully to these new conditions, the newborn’s microbiota has the highest plasticity compared to later in life. This state of highest susceptibility to the exposome and maximum of plasticity lasts up to two to three years and is termed ‘window of opportunity’ in our proposed model of the plasticity development of the human gut microbiota during the course of life (Figure 1). Yáñez-Ruiz et al. describe the microbial colonization of the rumen in the early stage of life and suggest possible ways of manipulation that could lead to lasting effects in the adult ruminant [104].

In summary, as soon as the newborn enters the new habitat outside the sterile maternal womb, it is immediately colonized by external microorganisms starting to establish its new individual human gut microbiota. This microbial composition is still very fragile and highly susceptible to environmental influences and has been associated with long-term health and disease outcomes.

### 2.3. Postnatal Period

During the postnatal period, important components of the exposome that imprint the microbiota remain infections as well as infant feeding mode with breastfeeding fostering beneficial microbe establishment in the gut. The first meals in an infant’s life ideally consist of breastmilk from its own mother, as it best matches the infant’s nutritional needs. Breast milk contains, next to lactose and fat, large amounts of human milk oligosaccharides (HMOs), which crucially participate in the maturation of the microbiota in the infant’s gut functioning as prebiotics [105]. Consumption of breast milk maintains the microbiota in the infant’s gut in a state of low diversity dominated by *Bifidobacterium* spp. This is beneficial to the infant’s health because the dominance of these bacteria in the gut reduces colonization by pathogens and ensures a healthy intestinal microbiota that reduces the risk of dangerous intestinal infections [103]. Breastfeeding could also prevent the onset of allergies later in life by directly shaping the newborn’s gut microbiota by exposure to the milk microbiota (10^4^–10^6^ bacterial cells per day [106]) as well as indirectly through milk factors such as HMOs, immunoglobulin A (IgA) and antimicrobial factors that affect bacterial growth [107].

Compared to consuming breast milk, infants consuming formula display a greater diversity, in this case suggesting a less stable and uniform population of the gut microbiota, making them more susceptible to infections [108,109]. Maternal nutrition also plays a crucial role in this developmental stage, as absence of nutrients or malnutrition in general has drastic effects not only on the mother herself, but also on the composition of the breast milk, which is associated with reduced levels of HMOs [19].

A major shift in the gut microbiota composition takes place at weaning, as the microbial community in the infant’s gut changes drastically after the introduction of solid foods, which usually occurs between 6 and 24 months of age [24]. Adaptation to diverse nutritional compounds leads to an increase in bacterial diversity and an increase in Lachnospiraceae and Ruminococcaceae [103]. Any dietary exposures during this early phase may impact the establishment of the microbiota and has been suggested to explain the development of asthma [110]. The Canadian Healthy Infant Longitudinal Development (CHILD) Study suggests that gut microbial dysbiosis in the first 100 days of life correlates with an increased risk of developing childhood asthma [111]. Increased asthma risk might be associated with a lack of microbial stimulation of the infant immune system within the first year of life [112]. Colonization with a low immunostimulatory microbiota during the first years of life may disrupt immune education, which can lead to inflammatory diseases and food allergies [113,114,115].

In summary, recent studies have suggested that a turning point in microbiota plasticity is reached at around 2 years of age, when the microbiota is largely established and components of the gut microbiota have been permanently imprinted by the previously experienced exposome. Therefore, these first years of life represent a ‘window of opportunity’ for microbial modulation [103]. The ‘first 1000 days’, in particular the period from birth to 2 years of age, is generally considered to be especially important for the healthy development of the gut microbiota of infants [103]. During this phase, the microbiota becomes a marker of the previously experienced exposome.

### 2.4. Childhood and Adolescence

There are few studies with controversial data comparing the composition of the gut microbiota during childhood and adolescence with that of adults. While some studies claim no difference between these groups, few studies describe a distinct pediatric gut microbiota. The KOALA Birth Cohort Study, a prospective Dutch cohort, has demonstrated that the overall gut microbiota of children aged 6 to 9 years has adult-like characteristics [116]. In contrast, other studies show that although the gut microbiota of healthy children already displays some adult-like patterns it seems to go through a longer developmental phase than previously anticipated, exhibiting its own distinct pediatric gut microbiota features [117]. The microbial community of the gut of a healthy child harbors significantly higher amounts of Firmicutes and Actinobacteria as well as lower amounts of Bacteroidetes compared to the adult microbiota [118]. Zhang et al. investigated fecal samples from 314 healthy young adults across China and defined a core gut microbiota consisting of bacterial genera describing their role in human health [119].

In summary, the pediatric gut microbiota displays some unique features, but mainly is already very similar to that of adult individuals. Although the microbiota has already become relatively stable, environmental factors and lifestyle still have an impact on the microbial composition. Events in childhood and adolescence can still have important effects on the microbiota, as some plasticity remains, but the microbiota is much less malleable than in earlier years of life.

### 2.5. Adulthood

After reaching adulthood, the interactions between the human host and its microorganisms in the gut stabilize and reach a homeostatic balance that reduces the response to environmental insults such as pathogenic infections [120,121,122]. During this phase of microbial development, a stable functional core microbiota is established with reduced intraindividual variation that appears to be independent of gender, geographic location, and age, while at the same time a large interindividual variation persists, creating a unique personal microbiota [123,124].

Nevertheless, this homeostasis can still be affected by drastic changes in diet, by infections that cause diarrhea, for example [125], and by the use of drugs such as antibiotics. However, Segal and Elinav suggest that in healthy individuals, the original microbial composition of the gut is largely restored one month after cessation of antibiotic treatment [126]. Interestingly, probiotic intake seems to induce a delayed and incomplete recovery of the microbiota after one month following antibiotic treatment. Other studies suggest periods of up to several months or even years before a person’s original microbiota is reestablished [127,128,129,130]. Although in some cases minor alterations may remain permanently [131], these changes might lead to an alternative stable state [132]. Nevertheless, the ultimate impact of such a shift remains unknown. In contrast, autologous fecal microbiota transplantation (FMT) restores the gut microbiota within a few days after antibiotic administration [126]. FMT from healthy donors proved highly effective in treating individuals with *Clostridioides difficile* infection, with beneficial effects lasting up to more than one year [133,134].

In summary, the exposome throughout adulthood can still affect the microbiota. In particular, extreme challenges, such as those induced by FMT, can have a lasting impact on the microbial composition. However, the microbiota is now largely determined and has become a mediator of the effects of the exposome on human health and disease. The microbiota may be temporarily malleable in adulthood as long as exposure continues but returns to its original and individual stage after the exposure ends, protecting the human host from environmental insults and infections, but also allowing adaptations to changing environmental stimuli.

### 2.6. Advanced Age

In general, advancing age leads to a decrease in physical fitness and an increased susceptibility to infections due to irreversible changes in the immune system leading to chronic low-grade inflammation or ‘inflammaging’. This frailty can lead to dysbiosis in the gut microbiota, which can cause pathogen overgrowth and disease onset, and is associated with a decrease in alpha diversity [135,136,137]. Several studies suggest that the human gut’s microbial composition changes continuously with increasing age of the host [136,138]. The onset of such degeneration of the microbiota is often observed in individuals above the age of 70 years, but studies mostly do not distinguish between healthy and diseased individuals [139].

A cohort study of 63 participants from northern Italy including young adults (20–40 years old), elderly (60–80 years old), as well as centenarians (99–104 years old), representing the average health status of the respective population group, found that the microbiota of the young adults and the elderly were very similar in terms of microbial composition and diversity measurements. The centenarian group on the other hand displayed a lower diversity and a different microbial composition with a higher abundance of pathobionts as well as higher level of systemic inflammation compared to the two younger groups [140]. A recent study analyzing the gut microbiota of 176 Korean adults aged 70 years and older links frailty measures to the *Bacteroides* enterotype with increased abundances of *Bacteroides fragilis* and *Clostridium hathewayi,* while beneficial bacterial taxa such as *Prevotella copri* and *Coprococcus eutactus* decreased. This suggests that the gut’s microbial composition serves as an indicator of an increased risk of frailty, but also provides a potential target for improving the health of frail elderly [141].

Nevertheless, the microbiota composition seems to correlate with the overall health status of the human host. In a cross-sectional study, over 1000 ‘extremely healthy’ Chinese individuals, ranging in age from 3 to over 100 years, with absence of any diseases and no personal and family history of cancers as well as cardiovascular, gastrointestinal, metabolic, respiratory, and neurological/mental diseases were studied, excluding up to 99% of the general population. In this study, Bian et al. observed that gut microbial composition of healthy elderly individuals who maintain a healthy lifestyle, including regular exercise and a balanced diet, did not differ much compared to healthy adults several decades younger. This suggests that healthy aging may be associated with the ability to maintain a ‘young’ microbiota [58].

In summary, maintaining a diverse gut microbiota with a microbial composition reflecting that of younger individuals appears to be associated with retaining a critical degree of plasticity and the prospect of healthy aging. Conversely, whether the potential to reach very old age is associated with the microbial state of the gut remains to be elucidated and requires further study.

## 3. Conclusions

In conclusion, the microbiota displays plasticity throughout the life course, with the maximum malleability and susceptibility during the perinatal and postnatal period, when the microbiota becomes established with a memory imprinted by the early exposome that may last a lifetime. The microbiota established during that time allows adequate responses to the vastly changing and challenging environmental insults and manifests as a marker of the exposome of early-life experiences. After the establishment of the microbiota, which is largely determined by the time the infant reaches 2 years of age, the microbiota becomes a mediator of future exposome exposures and their effects on human health. A ‘good’ microbiota is able to mediate negative influences while maintaining a good health status, while a ‘poor’ microbiota allows a harmful exposome to induce disease. Dysbiosis of the gut microbiota is associated with several diverse disease patterns and occurs more often with advancing age. Whether the physiological changes that occur with advancing age of the host lead to adaptation of the microbiota in the gut or whether the aging microbiota actively participates in the development of dysbiosis and resulting dysfunctions remains an important open question to be answered. The microbiota throughout adulthood may still be temporarily malleable to a certain extent, but retains a memory that mainly returns to the original stage after the exposure subsides. Other remaining questions include whether greater plasticity of the early microbiota predicts a long lifespan and/or healthy aging, and in what ways plasticity is associated with microbial diversity. Long-term observational studies are necessary to more precisely characterize the development of plasticity throughout the life course and to identify specific timeframes of vulnerability for intervention. In particular, the ‘window of opportunity’ at about 2 years of age, when the microbiota is most plastic and thus has the greatest chance to positively intervene and prevent the manifestation of a range of diseases later in life, should be investigated in more detail.

A better understanding of the plasticity of the microbiota, its memory, and the resulting characteristics that persist throughout life has important implications for the environment in the early years of life and for lifestyle in adulthood and could open new avenues for disease prevention. An early-established ‘good’ microbiota with the ability to maintain and restore microbial homeostasis might be conducive to healthy aging and longevity [142].

## Data Availability

Not applicable.
